# Sex-specific early cognitive changes are linked to global and pathway-specific genetic risk for Alzheimer’s disease in at-risk individuals

**DOI:** 10.1186/s13293-025-00800-w

**Published:** 2026-02-17

**Authors:** Patricia Genius, Alba Fernández-Bonet, Blanca Rodríguez-Fernández, Clara Gallay, Armand Gonzalez-Escalante, Gonzalo Sánchez-Benavides, David López-Martos, Manel Esteller, Arcadi Navarro, Juan D. Gispert, Anna Brugulat-Serrat, Natalia Vilor-Tejedor, the ALFA study

**Affiliations:** 1 Barcelonaβeta Brain Research Center (BBRC), Pasqual Maragall Foundation. C/Wellington, 30, Barcelona, 08005 Spain; 2 Centre for Genomic Regulation (CRG), Barcelona Institute of Science and Technology (BIST), C/Doctor Aiguader 88, Barcelona, 08003 Spain; 3 Hospital del Mar Medical Research Institute, Dr. Aiguader, 88, Barcelona, 08003 Spain; 4 Doctoral School, PhD programme in Bioinformatics, University of Vic–Central University of Catalonia (UVic-UCC), Vic, Spain; 5 Centro de Investigación Biomédica en Red de Fragilidad y Envejecimiento Saludable (CIBERFES), Instituto de Salud Carlos III, C/Monforte de Lemos 5, Pabellón 11, Madrid, 28029 Spain; 6 Centro de Investigación Biomédica en Red Cáncer (CIBERONC), C/Monforte de Lemos 5, Pabellón 11, Madrid, 28029 Spain; 7 Institució Catalana de Recerca i Estudis Avançats (ICREA), Passeig de Lluís Companys, 23, Barcelona, 08010 Catalonia Spain; 8 Camí de les Escoles, Josep Carreras Leukaemia Research Institute (IJC), Ctra de Can Ruti, Ctra. de Can Ruti, Camí de les Escoles s/n, Badalona, Barcelona, 08916 Spain; 9 Physiological Sciences Department, School of Medicine and Health Sciences, University of Barcelona (UB), Campus Clínic, Casanova, 143, Barcelona, 08036 Spain; 10 Institute of Evolutionary Biology (UPF-CSIC), Department of Experimental and Health Sciences, Universitat Pompeu Fabra, Passeig Marítim de la Barceloneta, 37-49, Barcelona, 08003 Spain; 11 Global Brain Health Institute, 675 Nelson Rising Lane, Suite 190, San Francisco, CA 94158 USA; 12 Department of Human Genetics, Radboud University Medical Center, Geert Grooteplein, Zuid 10, Nijmegen, 6525 GA The Netherlands

## Abstract

Alzheimer’s disease (AD) is a progressive neurodegenerative condition in which genetic predisposition plays a key role, yet the sex-specific mechanisms linking genetic risk to early cognitive changes remain unclear. This study examined the impact of polygenic risk scores (PRS) on early cognitive changes in 318 cognitively unimpaired participants from the ALFA+ cohort, a nested longitudinal cohort from the ALFA study (see details in Study Participants Section, Methods). Participants were followed for three years, with assessments across five cognitive domains and a preclinical composite (PACC). Global AD PRS, including and excluding the apolipoprotein E *(APOE)* gene, alongside five biologically informed pathway-specific PRS (amyloid, immune, external stimuli signaling, cholesterol efflux, lipoprotein metabolism) were computed. Generalized linear models including interaction by sex and stratified by sex and amyloid status (CSF Aβ42/40 < 0.071) assessed associations between PRS and cognitive change. In women, *APOE*-independent AD genetic risk predicted worse executive function, particularly via cholesterol efflux and external stimuli signaling pathways. Among Aβ + women, PRS also predicted lower memory performance, partially modulated by reproductive span. In Aβ − women, worse executive functioning performance was linked to amyloid, immune, and signaling pathways. In contrast, men showed associations between AD PRS and worse visual (Aβ−) and attentional (Aβ+) performance, independent of pathway-specific mechanisms. These findings reveal distinct, domain-specific cognitive vulnerabilities to AD genetic risk by sex and amyloid status, highlighting *APOE*-independent and mechanistic contributions to early and subtle cognitive changes. Results support the need for sex-aware, biologically informed genetic models in preclinical AD for risk stratification and early intervention.

**Supplementary Information:**

The online version contains supplementary material available at 10.1186/s13293-025-00800-w.

## Abstract (plain language summary)

Alzheimer’s disease (AD) is a condition that slowly damages the brain, affecting memory, thinking, and behavior over time. Some people are more likely to develop Alzheimer’s because of their genetic profile. One well-known gene, the *APOE gene*, increases AD genetic risk. Nonetheless, many other genes also play a role, and we still do not fully understand how these genes affect brain health, especially in the very early stages of the disease, before any symptoms appear.

In this study, we looked at 318 healthy adults who may be at higher genetic risk of AD. We followed their cognitive abilities over three years. We used genetic scores to estimate each person’s overall risk of developing AD and focused on specific genetic pathways related to processes like inflammation, cholesterol, and how brain cells communicate.

We found that women and men showed different patterns. In women, genetic risk (even without *APOE*) was linked to lower performance in specific cognitive abilities, such as memory and executive function. In women displaying AD-specific pathological burden in the brain, lower cognitive performance was stronger. In men, the genetic risk was tied to changes in visual and attention skills, but through different biological routes.

Our findings suggest that AD risk genes affect men and women differently, even before symptoms appear. Understanding these early, sex-specific patterns could help doctors predict who is at higher risk and lead to more personalized prevention strategies. This study highlights the importance of considering both sex and biology when studying AD.

**Supplementary Information:**

The online version contains supplementary material available at 10.1186/s13293-025-00800-w.

## Highlights

In Aβ− women, higher global AD genetic predisposition is associated with worse follow-up performance in episodic memory. The association is attenuated when *APOE* is included in the PRS and models are adjusted for reproductive span.

Across women, genetic predisposition to AD is associated with worse follow-up executive functioning performance, independently of *APOE*-ε4, amyloid status, and other confounders. Effects are primarily driven by cellular response to external stimuli.

In Aβ+ men, the association between genetic predisposition to AD and worse follow-up performance on attention is not independent of cardiovascular risk for dementia, and appears to be mainly driven by signaling pathways to external stimuli.

In Aβ− men, genetic predisposition to AD is associated with worse follow-up performance in visual processing independently of *APOE*-ε4 carriership, cardiovascular risk and neurodegeneration, although these associations are not attributable to any specific AD pathway.

**Supplementary Information:**

The online version contains supplementary material available at 10.1186/s13293-025-00800-w.

## Introduction

Alzheimer’s disease (AD) is a neurodegenerative disorder characterized by the progressive deterioration of cognitive abilities, including memory, language, and executive functions [1]. While substantial research has established the link between AD and cognitive impairment, the underlying genetic mechanisms contributing to early cognitive changes remain incompletely understood.

The apolipoprotein E *(APOE)* ε4 allele is the strongest known genetic risk factor for AD, associated with increased risk of progression from cognitively unimpaired (CU) to mild cognitive impairment (MCI) and dementia [2]. However, AD risk is polygenic [3, 4] and additional genetic variants, acting through *APOE*-related or independent mechanisms, contribute to disease vulnerability. These variants can be combined into polygenic risk scores (PRS), which estimate an individual’s cumulative genetic predisposition to AD [5]. PRS models have been increasingly used to assess genetic predisposition beyond *APOE* alone, providing a more comprehensive understanding of the broader genetic architecture of AD and its clinical heterogeneity [6, 7].

Emerging evidence suggests that higher PRS_AD_ is associated with cognitive decline even in CU individuals, years before symptoms emerge [8]. PRS_AD_ has also been linked to amyloid-beta (Aβ) pathology [9], hippocampal atrophy [10], and increased risk of conversion from CU to MCI and AD dementia [11, 12], supporting a role for genetic risk in early neurodegenerative processes.

More recently, AD pathway-specific PRS have emerged as valuable measures to assess how distinct biological mechanisms, such as amyloid processing, inflammation, and lipid metabolism, contribute to AD risk [13]. These measures allow for a mechanistic dissection of polygenic burden and its influence on cognitive decline.

Sex differences in AD have also been widely reported, with women exhibiting greater disease burden and faster cognitive decline than men [14]. While differences in longevity partially explain this, growing evidence points to underlying biological factors, such as hormonal changes, brain structure, and sex-specific gene-environment interactions [15, 16]. Women *APOE*-ε4 carriers show greater vulnerability to AD-related biomarkers, including increased amyloid deposition and neurodegeneration, compared to men carriers [17, 18]. However, whether broader genetic predisposition to AD is differentially associated with cognition in women and men remains underexplored. Given these sex differences in both genetic and clinical aspects of AD, it is crucial to investigate sex-specific genetic susceptibility to cognitive decline.

In this study, we investigated how genetic predisposition to AD, both global and pathway-specific, interacts with sex to shape domain-specific cognitive changes in CU individuals at risk for AD. We evaluated both AD and AD pathway-specific polygenic scores in global and *APOE*-independent PRS models, and tested whether associations varied by amyloid status. Furthermore, we explored whether these associations were independent of key biological factors, including reproductive span as a proxy for lifetime estrogen exposure, the Cardiovascular Risk Factors, Aging, and Incidence of Dementia I (CAIDE-I) score, and AD brain signature as an imaging marker of neurodegeneration.

## Methods

### Study participants

The study sample included 318 participants from the ALFA+ cohort. The ALFA+ cohort is a nested longitudinal study of the ALFA (for *Al*zheimer and *Fa*milies) parent cohort [19], composed of 2743 cognitively unimpaired (CU) individuals (between 45 and 75 years) enriched for family history of AD and genetic risk factors for AD [20]. The ALFA+ cohort was established in 2016 to provide detailed biomarker and cognitive follow-up assessments. Study participants were included based on available genetic data, fluid biomarkers and lifestyle risk factors, as well as detailed cognitive testing at baseline and follow-up [Supplementary Fig. 1]. Aβ pathology positivity (A+) was defined by CSF Aβ42/40 ratio (cutoff of positivity was < 0.071) [21]. Tau pathology positivity (T+) was determined by levels of CSF p-tau181 >0.24 pg/mL. The derived cut-offs defined AT groups (i.e. A-T-, A-T+, A + T-, A + T+). Individuals who were A-T+ (*n* = 11) were excluded because (i) by definition, A+ is required to meet the biological criteria for AD pathology and (ii) their number was too small to allow meaningful statistical inference. Sex was self-reported in ALFA+, and validated as a biological sex measure using genetic information, including heterozygosity on the X chromosome.

The ALFA+ study (ALFA-FPM-0311) was approved by the Independent Ethics Committee ‘Parc de Salut Mar’, Barcelona and registered at *Clinicaltrials.gov* (Identifier: NCT02485730). All participating subjects signed the study’s informed consent form, which was approved by the Independent Ethics Committee ‘Parc de Salut Mar’, Barcelona. The study was conducted according to the Declaration of Helsinki.

### Cognitive measures

Cognitive composite z-scores were computed in five different cognitive domains: attention (WAIS-IV: Digit Span, WMS-IV: Symbol Span and TMT-A), episodic memory (Free and Cued Selective Reminding Test, Memory Binding Test, WMS-IV Logical Memory and NIH-toolbox Picture Sequence Memory test), executive function (TMT-B, Five Digits test, WAIS-IV Coding, WAIS-IV Matrix reasoning and NIH-toolbox Flanker Inhibition test), language (Semantic fluency), and visual processing (WAIS-IV Visual Puzzles and RBANS Judgment of line orientation). In addition, the Preclinical Alzheimer’s Cognitive Composite (PACC) was computed, including the Total Paired Recall (TPR) and Total Delayed Free Recall scores of the Memory Binding Test, the Coding subtest of WAIS-IV, and semantic fluency, as defined in previous works [22]. To create cognitive composites, several individual tests were combined to construct an overall score representing a particular cognitive domain of interest. Cognitive composites were computed by standardizing each raw score into a z-score. Cognitive change for each composite was calculated by subtracting the follow-up visit score (2019) from the baseline cognitive score (2016). Negative values indicate worse performance in the follow-up visit.

### Genetic data acquisition, genotyping, quality control, and imputation

DNA was obtained from blood samples of the ALFA parent cohort through a salting-out protocol. In total, 2686 participants were genotyped. Genotyping was performed with the Illumina Infinium Neuro Consortium (NeuroChip) Array (build GRCh37/hg19) [23]. Quality control procedure was performed using PLINK software. Imputation was performed using the Michigan Imputation Server with the haplotype Reference Consortium Panel (HRC r1.1 2016) [24] following default parameters and established guidelines. A full description of the genotyping, quality control and imputation procedures is available elsewhere [20].

### Genetic profiling of alzheimer’s disease

After the corresponding quality control procedure, 2527 individuals remained with available genotype data. Genetic predisposition to AD was assessed using polygenic risk scoring through PRSice version 2 [25]. The AD polygenic score (PRS_AD_) was calculated by selecting representative genetic variants per linkage disequilibrium (LD) block with an LD threshold of r^2^ >0.1 within a 250-kb window. We additionally computed the PRS by excluding all single nucleotide polymorphisms in proximity to the *APOE* locus (GRch37; 19:45116911:46318605) referred to as PRS_ADno*APOE*_. Finally, we computed pathway-specific AD polygenic risk scores (pathPRS). Annotation of the significant single nucleotide polymorphisms (SNPs) (threshold of inclusion including < 10^− 6^) as well as enrichment analysis of the associated genes were performed using the *snpXplorer* web-server [26]. Based on gene overrepresentation, this analysis identified five biological pathways (immune system, external stimuli signaling, amyloid mechanisms, complex lipoprotein metabolism and cholesterol efflux). To evaluate the contribution of specific biological mechanisms, we computed pathway-specific PRSs by including SNPs associated with genes in each pathway (immune system, n_SNPs_=111; external stimuli signaling, n_SNPs_=104; amyloid mechanism, n_SNPs_=56; complex lipoprotein metabolism, n_SNPs_=29; cholesterol efflux, n_SNPs_=73) [Supplementary Table 1].

### Assessment of additional risk factors

Cardiovascular risk factors for dementia (body mass index, systolic blood pressure, age, sex, education, total cholesterol and physical activity) were included in the Cardiovascular Risk Factors, Aging, and Incidence of Dementia I (CAIDE-I) risk score predicting 20-year risk for late-life dementia in the baseline visit (2016). In the standard protocol for the CAIDE score computation [27], variables are dichotomized or categorized in tertiles based on standard cutoffs. In ALFA, participants were assigned a score depending on the group they belonged to for each one of the vascular risk factors [28].

Neuroimaging data was acquired for a subset of participants using magnetic resonance imaging (MRI) with a 3-T scanner (Ingenia CX, Philips, Amsterdam, Netherlands). All participants followed the same MRI protocol, which included a high-resolution 3D T1-weighted turbo field echo (TFE) sequence (voxel size: 0.75 × 0.75 × 0.75 mm, TR/TE: 9.90/4.6 ms, flip angle = 8°). Structural T1-weighted images were processed and segmented using FreeSurfer version 6.0 [29]. The AD brain signature was calculated by averaging the cortical thickness of specific brain regions known to be particularly vulnerable to AD-related degeneration. These regions included the medial temporal, inferior temporal, temporal pole, superior parietal, precuneus, angular, supramarginal, superior frontal, and middle frontal cortices [30]. Reproductive span, defined as the interval between menarche and menopause, was used as a proxy for cumulative endogenous estrogen exposure, given its association with hormone-sensitive processes over the life course.

### Statistical analysis

Differences in cognitive composite scores across sex, amyloid status and genetic predisposition to AD were assessed using χ2 tests for categorical variables, one-way ANOVA for normally distributed continuous variables, and non-parametric Kruskal-Wallis tests for non-normally distributed continuous variables. To evaluate the association between cognitive change and genetic predisposition to AD, sex- and sex/Aβ-stratified, generalized linear models were employed. Potential effect modification by sex and sex/Aβ status was explored through two-way (PRS*sex) and three-way (PRS*sex*Aβstatus) interaction terms. Models were adjusted by chronological age, years of education, and time between cognitive visits. To assess robustness of the findings, sensitivity analyses were performed, adjusting for potential confounders including the AD Dickerson brain signature, the CAIDE-I risk score, AT status, and reproductive span, in women. Statistical significance was set at *P* ≤ 0.05, with pairwise correction for group comparisons and adjustment for the false discovery rate (FDR). All significant interactions between sex and the global PRS of AD on predicting domain-specific cognitive changes, were additionally evaluated using the pathPRS. All statistical analyses and data visualizations were carried out using R and Biorender.

## Results

### Descriptive

The median age of the study’s participants at baseline was 61.3 [IQR: 57.7–64.5], 58.8% were women, and 34.3% were A+ [Table 1]. Women were significantly younger (*p* = 0.021), had lower educational attainment (*p* < 0.001), and a lower proportion of *APOE-ε*4 carriers (*p* = 0.027) than men. Moreover, women had a higher median value for AD Dickerson signature (*p* = 0.009) but a lower median CAIDE-I score (*p* < 0.001). At baseline, women outperformed men in memory tasks (*p* = 0.007), while men performed better in attention (*p* < 0.001), executive function (*p* = 0.002), and visual domains (*p* < 0.001) tasks. After 3 years, men showed a greater decline in attention (*p* < 0.001). Further characteristics of the sample and the distribution of potential risk factors according to amyloid status and sex are shown in Supplementary Table 2. Aβ + women were older than Aβ- women(*p* = 0.012), and a greater proportion of Aβ + women were *APOE*-ε4 carriers (71%) compared to Aβ- women (29%). Additionally, at baseline, Aβ + women exhibited significantly worse memory performance (*p* = 0.031) and PACC (*p* = 0.044). At follow-up, both Aβ + men and women showed worse cognitive performance than their Aβ- counterparts, particularly women in executive function (*p* = 0.045), and men in PACC (*p* = 0.001).

**Table 1:** Participants’ characteristics according to sex

	Total (*N* = 318)	Men (*N* = 131)	Women (*N* = 187)	*p*-value
***Demographics***				
Sex				
*Women*	187 (58.8%)			
*Men*	131 (41.2%)			
Age (median, IQR)	61.3 [57.7;64.5]	62.6 [59.0; 64.9]	60.1 [56.5; 64.2]	**0.021**
Years of education (median, IQR)	13.0 [11.0;17.0]	16.0 [12.0; 17.0]	12.0 [10.0; 17.0]	**< 0.001**
*APOE*-ε4 status (n, %)				**0.027**
*Non-carriers*	146 (45.9%)	50 (38.2%)	96 (51.3%)	
*Carriers*	172 (54.1%)	81 (61.8%)	91 (48.7%)	
Amyloid status				0.923
*Aβ42/40 -*	209 (65.7%)	87 (66.4%)	122 (65.2%)	
*Aβ42/40 +*	109 (34.3%)	44 (33.6%)	65 (34.8%)	
Tau profile				0.402
*T-*	282 (88.7%)	119 (90.8%)	163 (87.2%)	
*T+*	36 (11.3%)	12 (9.16%)	24 (12.8%)	
AT profile				0.796
*A-T-*	198 (62.3%)	83 (63.4%)	115 (61.5%)	
*A + T-*	84 (26.4%)	36 (27.5%)	48 (25.7%)	
*A-T+*	11 (3.46%)	4 (3.05%)	7 (3.74%)	
*A + T+*	25 (7.86%)	8 (6.11%)	17 (9.09%)	
AD brain Dickerson Signature	2.45 [2.39; 2.50]	2.44 [2.37; 2.49]	2.46 [2.40; 2.51]	**0.009**
CAIDE-I	6.00 [5.00; 8.00]	7.00 [5.00; 8.00]	6.00 [4.00; 7.00]	**< 0.001**
***Cognitive composites***,*** baseline (median***,*** IQR)***				
*Attention*	0.07 [−0.46; 0.51]	0.25 [−0.20; 0.69]	−0.18 [−0.69; 0.34]	**< 0.001**
*Memory*	0.08 [−0.38; 0.49]	−0.07 [−0.40; 0.29]	0.16 [−0.35; 0.59]	**0.007**
*Executive function*	0.02 [−0.50; 0.54]	0.16 [−0.30; 0.62]	−0.10 [−0.64; 0.48]	**0.002**
*Language*	0.18 [−0.59; 0.95]	0.18 [−0.59; 0.95]	−0.01 [−0.78; 0.76]	0.288
*Visual*	0.02 [−0.57; 0.64]	0.41 [−0.06; 0.92]	−0.19 [−0.85; 0.37]	**< 0.001**
*PACC*	0.09 [−0.39; 0.48]	0.11 [−0.23; 0.45]	0.04 [−0.45; 0.51]	0.354
***Cognitive composites***,*** rate of change (median***,*** IQR)***				
*Attention*	0.05 [−0.26; 0.38]	−0.03 [−0.35; 0.20]	0.16 [−0.17; 0.46]	**< 0.001**
*Memory*	0.02 [−0.22; 0.27]	0.10 [−0.24; 0.27]	−0.03 [−0.21; 0.27]	0.801
*Executive*	−0.03 [−0.33; 0.23]	−0.05 [−0.36; 0.11]	0.03 [−0.28; 0.30]	0.059
*Language*	−0.19 [−0.58; 0.58]	−0.19 [−0.77; 0.48]	0.00 [−0.58; 0.58]	0.487
*Visual*	0.05 [−0.36; 0.45]	0.00 [−0.36; 0.49]	0.05 [−0.37; 0.45]	0.739
*PACC*	0.03 [−0.22; 0.29]	0.01 [−0.29; 0.30]	0.05 [−0.19; 0.28]	0.201
Difference between time of cognitive visit (median, IQR)	3.15 [2.97; 3.44]	3.12 [2.93; 3.44]	3.17 [3.01; 3.45]	0.331
***AD polygenic risk scores (median***,*** IQR)***				
*PRS*_*AD*_	−0.03 [−0.16; 0.11]	−0.02 [−0.13; 0.16]	−0.04 [−0.18; 0.10]	0.073
*PRS*_*AD*_ * (excluding APOE region)*	−0.12 [−0.19; −0.01]	−0.11 [−0.18; −0.02]	−0.14 [−0.20; 0.01]	0.446
***Pathway-specific AD polygenic risk scores (median***,*** IQR)***				
*Amyloid pathway*	−0.02 [−0.18; 0.16]	0.00 [−0.16; 0.20]	−0.03 [−0.19; 0.13]	0.246
*Cholesterol efflux pathway*	0.12 [−0.13; 0.39]	0.21 [−0.02; 0.46]	0.06 [−0.19; 0.34]	**0.002**
*Stimulus pathway*	−0.12 [−0.28; 0.03]	−0.16 [−0.33; 0.04]	−0.11 [−0.26; 0.02]	0.399
*Immune pathway*	−0.32 [−0.48; −0.15]	−0.34 [−0.51; −0.17]	−0.31 [−0.45; −0.15]	0.336
*Complex lipoprotein pathway*	−0.22 [−0.61; 0.29]	−0.10 [−0.52; 0.39]	−0.29 [−0.68; 0.13]	**0.028**
*Amyloid pathway (excluding APOE region)*	0.18 [−0.03; 0.43]	0.23 [0.02; 0.46]	0.16 [−0.06; 0.41]	0.147
*Cholesterol efflux pathway (excluding APOE region)*	0.40 [0.06; 0.75]	0.50 [0.14; 0.78]	0.38 [0.03; 0.73]	0.067
*Stimulus pathway (excluding APOE region)*	−0.10 [−0.27; 0.03]	−0.11 [−0.30; 0.06]	−0.09 [−0.23; 0.02]	0.517
*Immune pathway (excluding APOE region)*	−0.23 [−0.42;0.00]	−0.25 [−0.48; <0.001]	−0.21 [−0.40; 0.01]	0.283
*Complex lipoprotein pathway (excluding APOE region)*	−0.05 [−0.84;0.02]	−0.05 [−0.86; 0.40]	−0.05 [−0.84; 0.02]	0.821

Amyloid positivity (Aβ+) was defined as CSF-Aβ42/40 < 0.071. Tau positivity (T+) was defined as CSF-pTau > 24 mg/dL. Footnote: information for AT profile and Tau profile is available for 307 individuals (11 A-T + individuals were excluded for the analyses working with CSF pTau levels). P-values correspond to F-test (continuous variables) and χ2 test (categorical variables). *p < 0.05

Amyloid positivity (Aβ+) was defined as CSF-Aβ42/40 < 0.071. Tau positivity (T+) was defined as CSF-pTau > 24 mg/dL. Information for AT profile and Tau profile is available for 307 individuals (11 A-T + individuals were excluded for the analyses working with CSF pTau levels). *P*-values correspond to F-test (continuous variables) and χ2 test (categorical variables). Significant *p*-values (*p < 0.05) are highlighted in bold

No significant sex differences were found in the PRS_AD_ [Supplemental Fig. 2]. Among pathway-specific scores, men showed higher cholesterol efflux scores (*p* = 0.024), though these differences were not present in *APOE* excluded models. Complex lipoprotein metabolism also displayed nominally higher scores in men. When stratified by amyloid status, Aβ+ women presented higher PRS_AD_, cholesterol efflux (*p* = 0.009), and complex lipoprotein metabolism scores (*p* = 0.037) than Aβ- women. In contrast, Aβ+ men showed significantly higher cholesterol efflux (*p* = 0.019) and complex lipoprotein metabolism scores (*p* = 0.019) compared to Aβ- men. These sex differences were not observed when *APOE*-related variants were excluded.

### Sex-dependent effect on the association between the genetic predisposition to AD and cognitive change

**Fig. 1:**
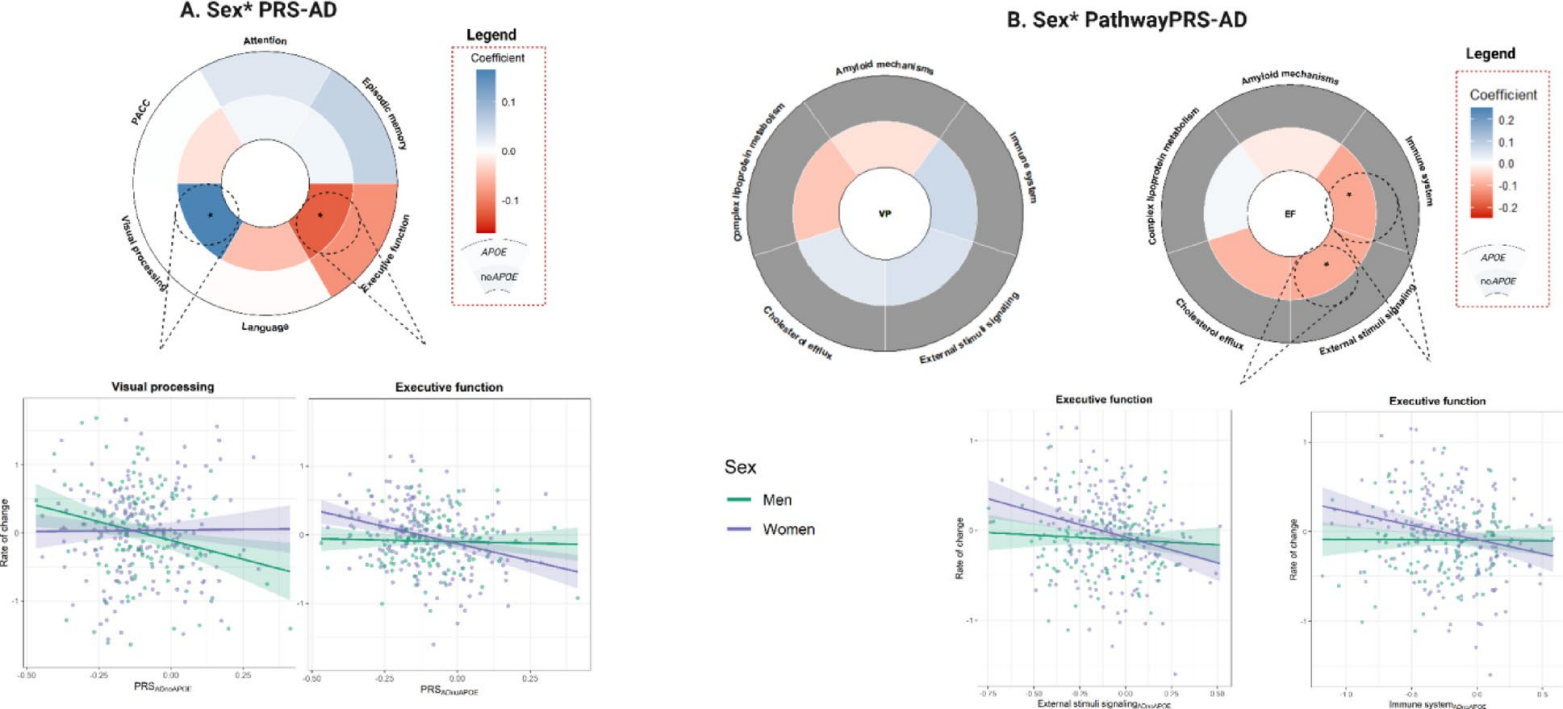
Results of the association models between (**A**) global PRSs and changes in cognitive performance adding an interaction term with sex. (**B**) Results with pathway-specific PRS were reported for significant results in (**A**). *Footnote*: Significant results are displayed (*:nominal p-value < 0.05; †:FDR p-value < 0.05). Blue indicates positive interaction term while red indicates negative one. Coefficients for the genetic scores including *APOE* variants are shown in the outer layer, while those excluding them are shown in the inner layer. For the results with the pathway-specific PRS, cells in gray indicate that the models with the pathway PRS, including *APOE* were not explored, as the global models only reported significant results for the PRS of AD when excluding the *APOE* variants.

Significant PRS-by-sex interactions were observed for the global PRS_AD_, when the *APOE* region was excluded, with executive function (p_interaction_ = 0.013) and visual domains (p_interaction_ = 0.034) as outcome variables [Figure 1A, Supplementary Table 3]. The PRS-by-sex significant interactions with executive function as an outcome did not remain significant after adjusting for *APOE*-ε4 carriership [Supplementary Table 4]. Results exploring pathPRS-by-sex interactions showed that sex modified the effect of the association between higher genetic risk of AD via external stimuli and immune system pathways, and executive functioning [Figure 1B, Supplementary Table 5]. No significant three-way (PRS*sex*Aβstatus) interactions were found [Supplemental Results].

**Fig. 2:**
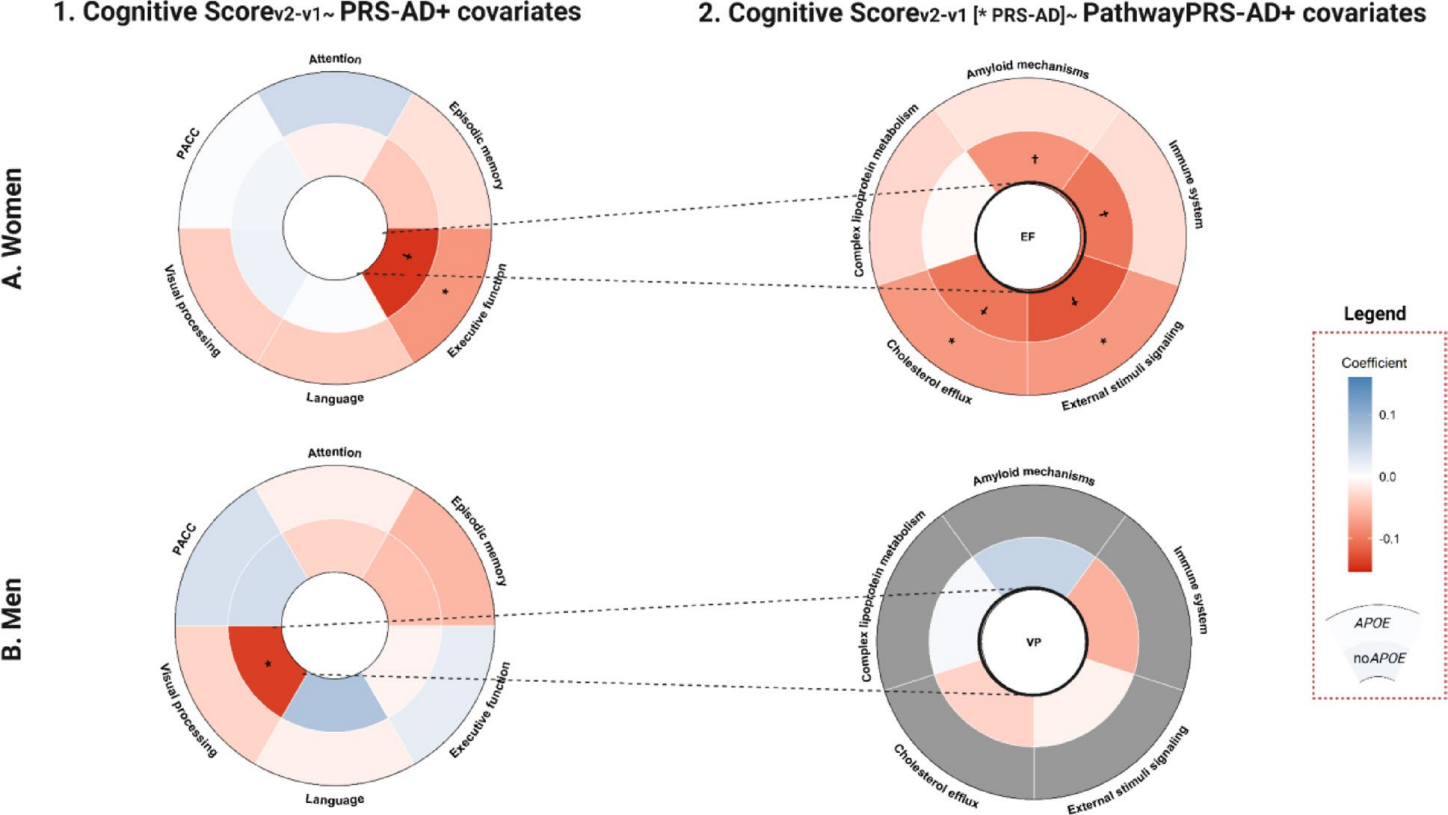
Results of the sex-stratified (women (**A**), men (**B**)) models exploring the association between (1) global and (2) pathway-specific PRSs and changes in cognitive performance. *Footnote*: Significant results are displayed (*: nominal p-value < 0.05; †:FDR p-value < 0.05). Blue indicates positive effect size while red indicates negative one. Coefficients for the genetic scores including *APOE* variants are shown in the outer layer, while those excluding them are shown in the inner layer. For the results with the pathway PRS, cells in gray indicate that the models with the pathway PRS including *APOE* were not explored, as the global models only reported significant results for the PRS of AD when excluding the *APOE* variants

In sex-stratified models, higher AD genetic risk was associated with worse executive function performance in women (ꞵ_PRSAD_ = −0.079, *p* = 0.026; ꞵ_PRSADno*APOE*_ = −0.144, *p* < 0.0001) [Figure 2A-1; Table 2]. Results remained significant after adjusting for potential confounders [Supplementary Table 6]. In women, AD genetic risk was associated with worse executive function, particularly via stimulus signaling (ꞵ = −0.077, *p* = 0.03) and cholesterol-related pathways (ꞵ = −0.077, *p* = 0.04) [Figure 2A-2, Supplementary Table 7]. These associations were more robust when *APOE*-related variants were excluded.

**Table 2:** Results of the generalized linear models for the association between the genetic predisposition to AD and changes in cognitive performance stratifying by sex.

Model	Women										Men									
	*N*	ꞵ	SE	T	*P*	CI lower	CI upper	*R* ^2^	Adj.*R*^2^	*P*.adj	*N*	ꞵ	SE	T	*P*	CI lower	CI upper	*R* ^2^	Adj.*R*^2^	*P*.adj
Change Attention ~ PRS_AD_ + Age + Education + Time_Visits + Aꞵ status	180	0.045	0.043	1.055	0.293	−0.039	0.13	0.047	0.020	0.879	127	−0.012	0.047	−0.264	0.792	−0.104	0.08	0.102	0.065	0.864
Change Attention ~PRS_ADno*APOE*_ + Age + Education + Time Visits + Aꞵ status	180	−0.011	0.042	−0.270	0.788	−0.095	0.072	0.041	0.014	0.946	127	−0.032	0.044	−0.721	0.472	−0.118	0.055	0.105	0.068	0.808
Change Memory ~ PRS_AD + Age + Education + Time Visits + Aꞵ status	180	−0.023	0.029	−0.800	0.425	−0.081	0.034	0.048	0.021	0.912	127	−0.054	0.036	−1.515	0.132	−0.125	0.017	0.051	0.011	0.612
Change Memory ~ PRS_ADnoAPOE + Age + Education + Time Visits + Aꞵstatus	180	−0.041	0.029	−1.426	0.156	−0.098	0.016	0.056	0.029	0.624	127	−0.048	0.034	−1.436	0.153	−0.115	0.018	0.049	0.010	0.612
Change Executive ~ PRS_AD + Age + Education + Time Visits + Aꞵ status	180	−0.079	0.035	−2.239	**0.026**	−0.149	−0.009	0.058	0.031	0.156	127	0.022	0.036	0.616	0.539	−0.049	0.093	0.082	0.044	0.808
Change Executive ~ PRS_ADnoAPOE + Age + Education + Time Visits + Aꞵstatus	180	−0.144	0.034	−4.271	**0.000**	−0.211	−0.078	0.123	0.098	**0.000**	127	−0.009	0.034	−0.282	0.779	−0.076	0.057	0.080	0.042	0.864
Change Language ~ PRS_AD + Age + Education + Time Visits + Aꞵstatus	180	−0.038	0.061	−0.626	0.532	−0.16	0.083	0.028	0.000	0.912	127	−0.011	0.085	−0.136	0.892	−0.179	0.156	0.017	−0.023	0.892
Change Language ~ PRS_ADnoAPOE + Age + Education + Time_Visits + Aꞵstatus	180	0.004	0.061	0.060	0.952	−0.117	0.124	0.026	−0.002	0.952	127	0.073	0.079	0.920	0.359	−0.084	0.23	0.024	−0.017	0.794
Change Visual ~ PRS_AD + Age + Education + Time Visits + Aꞵstatus	180	−0.036	0.049	−0.723	0.471	−0.133	0.062	0.024	−0.004	0.912	127	−0.032	0.065	−0.497	0.620	−0.16	0.096	0.083	0.046	0.827
Change Visual ~ PRS_ADnoAPOE + Age + Education + Time_Visits + Aꞵstatus	180	0.016	0.049	0.328	0.743	−0.08	0.112	0.021	−0.007	0.946	127	−0.139	0.060	−2.329	**0.022**	−0.257	−0.021	0.121	0.085	0.264
Change PACC ~ PRS_AD + Age + Education + Time Visits + Aꞵstatus	180	0.004	0.028	0.129	0.898	−0.052	0.059	0.037	0.010	0.952	127	0.037	0.043	0.850	0.397	−0.049	0.122	0.092	0.055	0.794
Change PACC ~ PRS_ADnoAPOE + Age + Education + Time Visits + Aꞵstatus	180	0.012	0.028	0.451	0.653	−0.042	0.067	0.038	0.011	0.946	127	0.040	0.041	0.973	0.333	−0.041	0.12	0.094	0.057	0.794

95% confidence intervals are reported. Significant *p*-values (*p < 0.05) are highlighted in bold

In men, higher AD genetic predisposition was associated with visual processing decline (ꞵ_PRSADno*APOE*_= −0.139, *p* = 0.022) [Figure 2B-1; Table 2]. Nonetheless, this association was neither independent of *APOE-*ε4 carriership [Supplementary Table 6] nor pathway-specific [Figure 2B-2, Supplementary Table 8].

### Sex-specific effect on the association between the genetic predisposition to AD and cognitive change across amyloid profiles

**Fig. 3:**
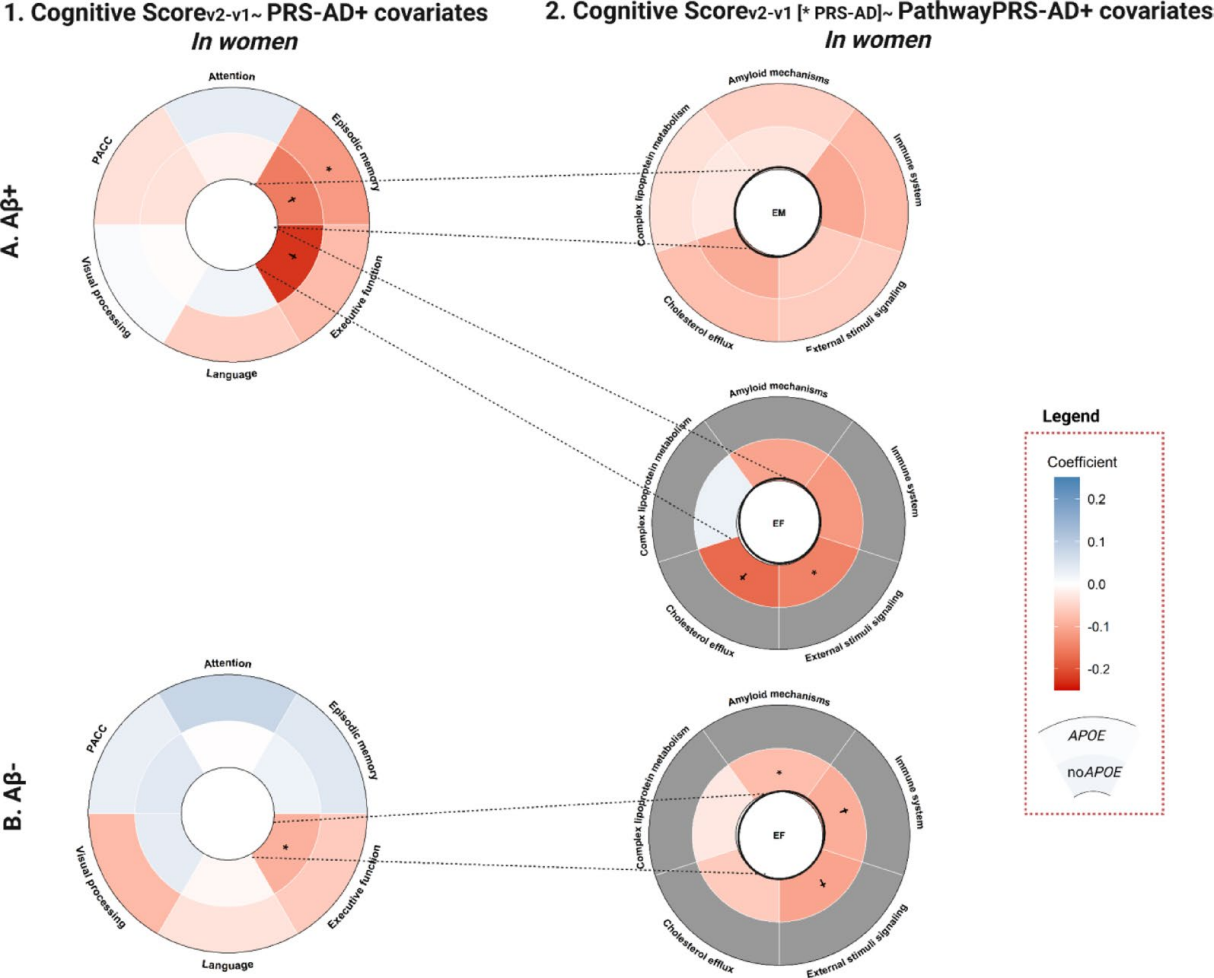
Results of the Aꞵ-stratified models in women (Aꞵ+ (**A**), Aꞵ- (**B**)), exploring the association between (1) global and (2) pathway-specific PRSs and changes in cognitive performance. *Footnote*: Significant results are displayed (*: nominal p-value < 0.05; † FDR p-value < 0.05). Blue indicates positive effect size while red indicates negative one. Coefficients for the genetic scores including *APOE* variants are shown in the outer layer, while those excluding them are shown in the inner layer. For the results with the pathway PRS, cells in gray indicate that the models with the pathway PRS including *APOE* were not explored, as the global models only reported significant results for the PRS of AD when excluding the *APOE* variants

In Aβ + women, higher AD genetic burden was associated with worse memory performance (ꞵ_PRSAD_ = −0.119, *p* = 0.037; ꞵ_PRSADno*APOE*_= −0.153, *p* = 0.007) [Figure 3A-1; Table 3]. The effect remained significant after excluding *APOE* variants, even after adjusting for *APOE-*ε4 carriership (ꞵ_PRSAD_ = −0.116, *p* = 0.042), but was no longer significant when adjusting for reproductive span (*p* = 0.181) [Supplementary Table 9 A]. Worse executive function performance in women, was associated with higher genetic risk of AD when excluding *APOE* variants, both in Aꞵ- and Aꞵ+ groups (Aꞵ- ꞵ_PRSADno*APOE*_ = −0.092, *p* = 0.017; Aꞵ+ ꞵ_PRSADno*APOE*_ = −0.225, *p* = 0.001) [Table 3]. In Aꞵ- women, the association did not remain significant after adjusting for reproductive span (*p* = 0.077) [Supplementary Table 9B]. Distinct pathways contributed to the explained variability of executive function worsening in women: cholesterol efflux (ꞵ_PathPRScholesterol_ = −0.174, *p* = 0.010) and stimulus signaling (ꞵ_PathPRScholesterol_ = −0.147, *p* = 0.029) in Aꞵ+ [Figure 3A-2, Supplementary Table 10A] and amyloid (ꞵ_PathPRSamyloid_ = −0.078, *p* = 0.046), stimulus signaling (ꞵ_PathPRSsignaling_ = −0.108, *p* = 0.005) and immune pathways (ꞵ_PathPRSimmune_ = −0.096, *p* = 0.015) in Aꞵ- [Table 3, Supplementary Table 10B].

**Table 3:** Results of the generalized linear models for the association between the genetic predisposition to AD and changes in cognitive performance stratifying by sex and Aβ status

		AB-									
Model	Sex	N	Beta	SE	T	P	CI lower	CI upper	R2	Adj.R2	P.adj
Change Attention ~ PRS_AD_ + Age+Education+Time Visits	Women	115	0.078	0.051	1.546	0.125	-0.022	0.179	0.063	0.029	0.412
Change Attention ~ PRS_ADno*APOE*_ + Age+Education+Time Visits	Women	115	-0.001	0.050	-0.029	0.977	-0.101	0.098	0.043	0.008	0.977
Change Memory ~ PRS_AD_ + Age+Education+Time Visits	Women	115	0.044	0.032	1.377	0.171	-0.019	0.107	0.056	0.021	0.412
Change Memory ~ PRS_ADno*APOE*_ + Age+Education+Time Visits	Women	115	0.023	0.031	0.722	0.472	-0.04	0.085	0.044	0.009	0.708
Change Executive ~ PRS_AD_ + Age+Education+Time Visits	Women	115	-0.061	0.039	-1.539	0.127	-0.139	0.017	0.051	0.016	0.412
Change Executive ~ PRS_ADno*APOE*_ + Age+Education+Time Visits	Women	115	-0.092	0.038	-2.425	**0.017**	-0.168	-0.017	0.079	0.046	0.204
Change Language ~ PRS_AD_ + Age+Education+Time Visits	Women	115	-0.033	0.076	-0.430	0.668	-0.184	0.119	0.023	-0.012	0.802
Change Language ~ PRS_ADno*APOE*_ + Age+Education+Time Visits	Women	115	-0.009	0.075	-0.114	0.910	-0.157	0.14	0.022	-0.014	0.977
Change Visual ~ PRS_AD_ + Age+Education+Time Visits	Women	115	-0.082	0.063	-1.294	0.198	-0.207	0.043	0.036	0.001	0.412
Change Visual ~ PRS_ADno*APOE*_ + Age+Education+Time Visits	Women	115	0.036	0.062	0.574	0.567	-0.088	0.159	0.025	-0.011	0.756
Change PACC ~ PRS_AD_ + Age+Education+Time Visits	Women	115	0.030	0.033	0.926	0.356	-0.035	0.095	0.017	-0.019	0.610
Change PACC ~ PRS_ADno*APOE*_ + Age+Education+Time Visits	Women	115	0.041	0.032	1.271	0.206	-0.023	0.104	0.023	-0.012	0.412

Model	Sex	N	Beta	SE	T	P	CI lower	CI upper	R2	Adj.R2	P.adj
Change Attention ~ PRS_AD_ + Age+Education+Time Visits	Men	83	0.046	0.068	0.687	0.494	-0.088	0.181	0.112	0.066	0.675
Change Attention ~ PRS_ADno*APOE*_ + Age+Education+Time Visits	Men	83	0.029	0.059	0.491	0.625	-0.089	0.147	0.109	0.063	0.750
Change Memory ~ PRS_AD_ + Age+Education+Time Visits	Men	83	-0.001	0.045	-0.017	0.986	-0.091	0.089	0.037	-0.012	0.988
Change Memory ~ PRS_ADno*APOE*_ + Age+Education+Time Visits	Men	83	-0.042	0.039	-1.071	0.287	-0.12	0.036	0.051	0.002	0.574
Change Executive ~ PRS_AD_ + Age+Education+Time Visits	Men	83	0.095	0.052	1.833	0.071	-0.008	0.198	0.070	0.022	0.426
Change Executive ~ PRS_ADno*APOE*_ + Age+Education+Time Visits	Men	83	0.031	0.046	0.668	0.506	-0.061	0.122	0.035	-0.014	0.675
Change Language ~ PRS_AD_ + Age+Education+Time Visits	Men	83	0.002	0.119	0.015	0.988	-0.234	0.238	0.011	-0.039	0.988
Change Language ~ PRS_ADno*APOE*_ + Age+Education+Time Visits	Men	83	0.129	0.103	1.259	0.212	-0.075	0.333	0.031	-0.019	0.509
Change Visual ~ PRS_AD_ + Age+Education+Time Visits	Men	83	-0.078	0.088	-0.884	0.379	-0.252	0.097	0.033	-0.016	0.650
Change Visual ~ PRS_ADno*APOE*_ + Age+Education+Time Visits	Men	83	-0.211	0.073	-2.885	**0.005**	-0.357	-0.065	0.118	0.073	0.060
Change PACC ~ PRS_AD_ + Age+Education+Time Visits	Men	83	0.088	0.059	1.503	0.137	-0.029	0.205	0.048	-0.001	0.471
Change PACC ~ PRS_ADno*APOE*_ + Age+Education+Time Visits	Men	83	0.073	0.051	1.428	0.157	-0.029	0.175	0.045	-0.004	0.471

		AB+									
Model	Sex	N	Beta	SE	T	P	CI lower	CI upper	R2	Adj.R2	P.adj
Change Attention ~ PRS_AD_ + Age+Education+Time Visits	Women	65	0.034	0.077	0.439	0.662	-0.121	0.189	0.059	-0.004	0.959
Change Attention ~ PRS_ADno*APOE*_ + Age+Education+Time Visits	Women	65	-0.015	0.078	-0.199	0.843	-0.171	0.14	0.057	-0.006	0.959
Change Memory ~ PRS_AD_ + Age+Education+Time Visits	Women	65	-0.119	0.056	-2.132	**0.037**	-0.231	-0.007	0.141	0.084	0.148
Change Memory ~ PRS_ADno*APOE*_ + Age+Education+Time Visits	Women	65	-0.153	0.055	-2.790	**0.007**	-0.262	-0.043	0.182	0.128	**0.042**
Change Executive ~ PRS_AD_ + Age+Education+Time Visits	Women	65	-0.079	0.069	-1.154	0.253	-0.216	0.058	0.092	0.032	0.759
Change Executive ~ PRS_ADno*APOE*_ + Age+Education+Time Visits	Women	65	-0.225	0.063	-3.566	**0.001**	-0.351	-0.099	0.234	0.183	**0.012**
Change Language ~ PRS_AD_ + Age+Education+Time Visits	Women	65	-0.056	0.107	-0.522	0.604	-0.271	0.159	0.041	-0.023	0.959
Change Language ~ PRS_ADno*APOE*_ + Age+Education+Time Visits	Women	65	0.020	0.108	0.190	0.850	-0.195	0.236	0.038	-0.027	0.959
Change Visual ~ PRS_AD_ + Age+Education+Time Visits	Women	65	0.007	0.077	0.095	0.925	-0.148	0.162	0.100	0.040	0.959
Change Visual ~ PRS_ADno*APOE*_ + Age+Education+Time Visits	Women	65	-0.004	0.078	-0.052	0.959	-0.159	0.151	0.099	0.039	0.959
Change PACC ~ PRS_AD_ + Age+Education+Time Visits	Women	65	-0.035	0.053	-0.659	0.513	-0.141	0.071	0.056	-0.007	0.959
Change PACC ~ PRS_ADno*APOE*_ + Age+Education+Time Visits	Women	65	-0.034	0.053	-0.642	0.523	-0.14	0.072	0.055	-0.008	0.959

Model	Sex	N	Beta	SE	T	P	CI lower	CI upper	R2	Adj.R2	P.adj
Change Attention ~ PRS_AD_ + Age+Education+Time Visits	Men	44	-0.121	0.061	-1.990	0.054	-0.245	0.002	0.227	0.148	0.324
Change Attention ~ PRS_ADno*APOE*_ + Age+Education+Time Visits	Men	44	-0.136	0.064	-2.121	**0.040**	-0.265	-0.006	0.237	0.159	0.324
Change Memory ~ PRS_AD_ + Age+Education+Time Visits	Men	44	-0.103	0.065	-1.586	0.121	-0.233	0.028	0.073	-0.022	0.484
Change Memory ~ PRS_ADno*APOE*_ + Age+Education+Time Visits	Men	44	-0.054	0.070	-0.771	0.445	-0.196	0.088	0.028	-0.072	0.831
Change Executive ~ PRS_AD_ + Age+Education+Time Visits	Men	44	-0.046	0.047	-0.989	0.329	-0.141	0.048	0.230	0.151	0.790
Change Executive ~ PRS_ADno*APOE*_ + Age+Education+Time Visits	Men	44	-0.052	0.049	-1.062	0.295	-0.152	0.047	0.233	0.154	0.790
Change Language ~ PRS_AD_ + Age+Education+Time Visits	Men	44	-0.025	0.132	-0.192	0.849	-0.293	0.242	0.013	-0.088	0.920
Change Language ~ PRS_ADno*APOE*_ + Age+Education+Time Visits	Men	44	-0.014	0.140	-0.101	0.920	-0.297	0.269	0.012	-0.089	0.920
Change Visual ~ PRS_AD_ + Age+Education+Time Visits	Men	44	0.070	0.100	0.706	0.485	-0.132	0.273	0.285	0.212	0.831
Change Visual ~ PRS_ADno*APOE*_ + Age+Education+Time Visits	Men	44	0.028	0.106	0.268	0.790	-0.186	0.243	0.278	0.204	0.920
Change PACC ~ PRS_AD_ + Age+Education+Time Visits	Men	44	0.016	0.069	0.230	0.819	-0.124	0.156	0.046	-0.051	0.920
Change PACC ~ PRS_ADno*APOE*_ + Age+Education+Time Visits	Men	44	0.014	0.073	0.184	0.855	-0.135	0.162	0.046	-0.052	0.920

95% confidence intervals are reported. Significant *p*-values (*p < 0.05) are highlighted in bold

**Fig. 4:**
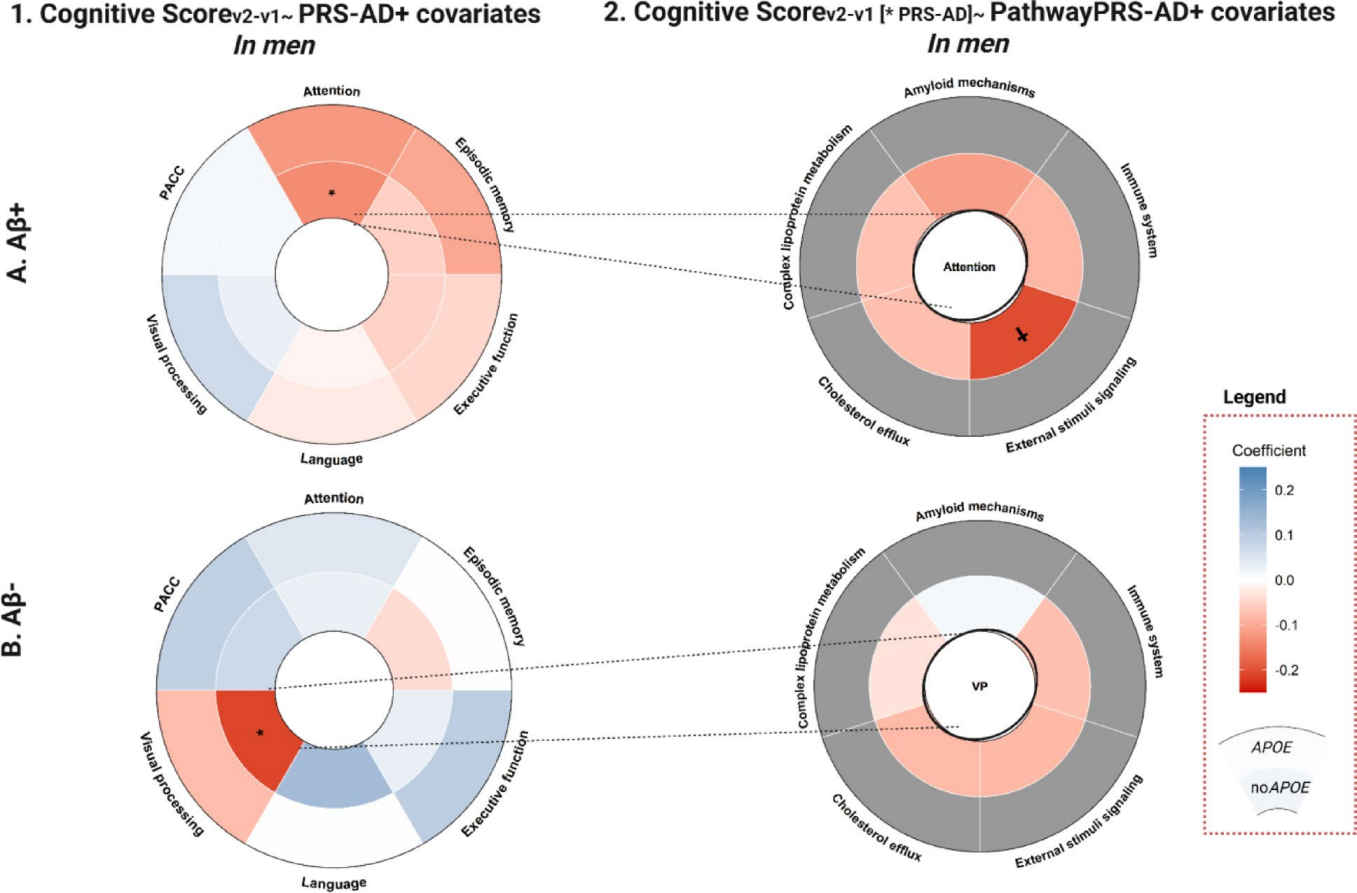
Results of the Aꞵ-stratified models in men (Aꞵ+ (**A**), Aꞵ- (**B**)), exploring the association between (1) global and (2) pathway-specific PRSs and changes in cognitive performance. *Footnote*: Significant results are displayed (*: nominal p-value < 0.05; †:FDR p-value < 0.05). Blue indicates a positive effect size while red indicates a negative one. Coefficients for the genetic scores including *APOE* variants are shown in the outer layer, while those excluding them are shown in the inner layer. For the results with the pathway PRS, cells in gray indicate that the models with the pathway PRS including *APOE* were not explored, as the global models only reported significant results for the PRS of AD when excluding the *APOE* variants

In Aβ + men, higher PRS_ADno*APOE*_ was associated with worse performance in attention (ꞵ_PRSADno*APOE*_ = −0.136, *p* = 0.04) [Figure 4A-1; Table 3], although this result was no longer significant after adjusting for the CAIDE-I risk score [Supplementary Table 11 A]. This association was particularly observed through the stimulus signaling-related pathway (ꞵ_PathPRSsignaling_=−0.204, *p* = 0.001) [Figure 4A-2, Supplementary Table 12 A]. In Aβ- men, higher PRS_ADno*APOE*_ was associated with worse performance in visual processing (ꞵ_PRSADno*APOE*_ = −0.211, *p* = 0.005) independent of confounding factors [Figure 4B-1, Supplementary Table 11B], but non-pathway specific [Figure 4B-2, Supplementary Table 12B].

## Discussion

Our study provided novel evidence that genetic predisposition to AD affects cognitive performance in a sex-dependent and domain-specific manner, even at early stages of the disease continuum. By integrating pathway-specific polygenic scores and examining both *APOE*-included and *APOE*-excluded models, we identified distinct associations between higher AD genetic risk and worse cognitive performance in executive function, memory, attention, and visual processing. These associations were often stronger in *APOE*-independent models and varied by sex and amyloid status, suggesting that both biological sex and non-*APOE* genetic burden shape the cognitive profile of early AD risk.

In women, higher genetic risk, particularly in *APOE*-independent models, was associated with worse performance in executive function. This effect was observed across amyloid groups. These findings align with prior work suggesting that women may be more vulnerable to early executive dysfunction in AD [31]. Previous studies have demonstrated that women exhibit steeper rates of cognitive decline following AD diagnosis and may experience different patterns of neurodegeneration compared to men, with greater vulnerability in frontal and executive networks [31]. Amyloid-stratified analyses provided additional mechanistic insights into these sex-specific effects. In Aβ+ women, AD genetic predisposition was explicitly associated with worse follow-up performance in episodic memory in a non-pathway specific manner, but with worse executive functioning via cholesterol efflux and stimuli signaling pathways. The involvement of cholesterol efflux pathways in this context is particularly noteworthy, given the well-established bidirectional relationship between cholesterol homeostasis and AD pathology [32]. Dysregulated cholesterol metabolism has been linked to increased amyloid-β production and impaired clearance, and cholesterol serves as a precursor for sex hormones, including estrogen, which exerts neuroprotective and anti-inflammatory effects through genomic and non-genomic mechanisms [33]. Given the regulatory role of estrogen on brain function and lipid homeostasis, disruption of these pathways may differentially affect cognitive decline in women [34]. In contrast, in Aβ- women, stimulus signaling, immune system and amyloid-related pathways were more strongly implicated in worse executive performance. This pattern suggests that in the absence of detectable amyloid pathology, genetic risk may manifest through alterations in synaptic signaling, neuronal communication, cellular reactivity and early amyloidogenic processes that precede plaque formation.

Altogether, these findings complement our earlier observation that worse follow-up performance in executive function was associated with AD genetic risk across amyloid groups, suggesting that while executive vulnerability is a consistent feature of genetic risk in women, the underlying biological mechanisms shift depending on amyloid status. However, the persistence of the stimuli signaling pathway across both amyloid states highlights the central role of neurons, microglia and astrocytes in overreacting to environmental or internal challenges (e.g. oxidative stress, metabolic changes). This overreactivity appears to occur independently of amyloid-specific mechanisms or disease stages, suggesting a continuous vulnerability to executive functioning.

Nonetheless, the associations between genetic risk of AD and executive functioning and memory, were attenuated when adjusting for reproductive span, suggesting that hormonal history may mediate or moderate the genetic effects on cognitive decline. It is important to note that reproductive span is used as a proxy for lifetime endogenous estrogen exposure. Longer reproductive span, reflecting earlier menarche and later menopause, has been associated with prolonged estrogen exposure and better cognitive outcomes [35]. These results emphasize the critical need to further investigate the complex interplay between sex hormones, reproductive history, and AD genetics in shaping preclinical cognitive trajectories, and highlight reproductive span as a potentially modifiable or targetable factor in understanding sex-specific AD risk.

Men, by contrast, demonstrated distinct associations between AD genetic burden and cognitive changes, particularly affecting the performance in visual processing and attention tasks. These genetic effects varied by amyloid status. In Aβ− men, elevated non-*APOE* AD genetic burden predicted worse performance in visual processing tasks. Non-specific pathways were driving this association. These findings may suggest the contribution of global AD burden to network disruption, specifically in occipito-parietal networks involved in visuospatial and attentional control [36]. Conversely, Aβ+ men showed significant associations between AD genetic risk and worse performance in attention, primarily via stimulus-signaling pathway disruption. In the presence of amyloid pathology, neuronal signaling may become disrupted and affect attention control networks.

These findings support the hypothesis that men and women follow distinct cognitive and neurobiological trajectories under genetic risk [37, 38]. The domain-specific vulnerabilities observed in men may reflect early susceptibility of posterior brain regions to tau pathology or posterior cortical atrophy. These manifestations are often underrecognized in typical AD models yet have been linked to visual processing impairment and may exhibit greater prevalence in men [39, 40].

Our findings also highlight the importance of disentangling *APOE* effects from the broader genetic architecture of AD. While *APOE*-ε4 is the primary driver of late-onset AD risk, its dominant effect may obscure contributions from other genetic loci in polygenic models. We observed stronger associations between PRS and cognition when *APOE* variants were excluded, particularly in women, suggesting that non-*APOE* variants exert independent cognitive effects that are biologically meaningful but difficult to detect when *APOE* dominates the polygenic signal. Prior studies have demonstrated that excluding *APOE* can unmask additional risk pathways operating through immune response, metabolic dysregulation, and other non-canonical mechanisms [39, 40]. Removing *APOE* thus provides clearer insight into the broader polygenic architecture underlying early and sex-specific cognitive vulnerability. Our results align with accumulating evidence that sex modifies the genetic influence on preclinical AD trajectories. Prior longitudinal studies in preclinical cohorts reported stronger associations between genetic risk and cognitive decline and resilience in women, consistent with our findings in executive and memory domains [41, 42]. The involvement of cholesterol efflux and immune pathways corroborates emerging molecular evidence implicating lipid metabolism and inflammation as sex-differentiated mechanisms in early AD pathogenesis [33].

To our knowledge, this is one of the first studies to jointly examine the interplay between sex, amyloid status, and AD genetic predisposition, incorporating pathway-specific risk models and proxies of hormonal exposure, in CU individuals. By integrating these factors, we uncovered domain-specific and sex-dependent patterns of early cognitive changes, revealing how distinct genetic pathways may differentially influence cognition across the early stages of the disease. These findings underscore the importance of considering both sex and genetic architecture in AD research and support the development of personalized prevention and treatment strategies.

Nonetheless, several limitations warrant consideration in this study. First, the relatively short follow-up period (3.2 years) and limited number of visits restrict our ability to draw firm conclusions about long-term cognitive trajectories. The modest magnitude of cognitive change observed is expected, given this timeframe; however, the identification of significant genetic associations despite this constraint supports the sensitivity of the ALFA+ design to capture early preclinical effects. This interpretation is supported by the 21% of participants reporting subjective cognitive decline [43], indicating early cognitive vulnerability consistent with the observed genetic effects. Ongoing data collection will enable more robust longitudinal modeling of cognitive trajectories.

Second, replicating our results in larger, more diverse cohorts will be essential to confirm these findings and address statistical power limitations. While our findings may not be directly generalizable to all populations, the ALFA+ cohort’s genetic and demographic enrichment provides a uniquely sensitive framework to detect subtle cognitive changes in at-risk individuals and preclinical AD stages. This design prioritizes mechanistic insight over population representativeness, thereby generating hypotheses about sex- and pathway-specific vulnerabilities that can guide replication in larger, more heterogeneous samples. Furthermore, our cohort excluded individuals with relevant medical conditions or neurological diseases, which could limit the generalizability of our results to broader populations. However, the lack of comorbidities and the younger age of our cohort represent strengths, as they allow for identifying subtle changes in cognitive function at early stages of the AD *continuum*.

In conclusion, our findings underscore the importance of considering sex-specific mechanisms in understanding AD genetic risk and cognitive decline. Women showed *APOE*-independent genetic burden associations with worse follow-up performance in executive function and memory, possibly influenced by hormonal and immune-metabolic pathways. In men, genetic predisposition was associated with worse follow-up performance in visual and attentional domains, mainly influenced by neuronal signaling and synaptic function, suggesting domain- and sex-specific vulnerability. These sex-specific patterns support the development of precision risk stratification models for individuals at risk for AD dementia, and emphasize the necessity to consider both sex and AD genetic risk profile for understanding early cognitive changes in at-risk populations.

## Supplementary Information

Supplementary Material 1

## Data Availability

No datasets were generated or analysed during the current study.

## References

[1] DeTure MA, Dickson DW. The neuropathological diagnosis of Alzheimer’s disease. Mol Neurodegener. 2019;14:32. 31375134 10.1186/s13024-019-0333-5 PMC6679484

[2] Corder EH, Saunders AM, Strittmatter WJ, Schmechel DE, Gaskell PC, Small GW, et al. Gene dose of apolipoprotein E type 4 allele and the risk of Alzheimer’s disease in late onset families. Science. 1993;261:921–3. 8346443 10.1126/science.8346443

[3] de Rojas I, Moreno-Grau S, Tesi N, Grenier-Boley B, Andrade V, Jansen IE, et al. Common variants in Alzheimer’s disease and risk stratification by polygenic risk scores. Nat Commun. 2021;12:3417. 34099642 10.1038/s41467-021-22491-8 PMC8184987

[4] Lambert J-C, Ramirez A, Grenier-Boley B, Bellenguez C. Step by step: towards a better understanding of the genetic architecture of Alzheimer’s disease. Mol Psychiatry. 2023;28:2716–27. 37131074 10.1038/s41380-023-02076-1 PMC10615767

[5] Lewis CM, Vassos E. Polygenic risk scores: from research tools to clinical instruments. Genome Med. 2020;12:44. 32423490 10.1186/s13073-020-00742-5 PMC7236300

[6] Stocker H, Möllers T, Perna L, Brenner H. The genetic risk of Alzheimer’s disease beyond APOE ε4: systematic review of Alzheimer’s genetic risk scores. Transl Psychiatry. 2018;8:166. 30143603 10.1038/s41398-018-0221-8 PMC6109140

[7] Skoog I, Kern S, Najar J, Guerreiro R, Bras J, Waern M, et al. A non-APOE polygenic risk score for Alzheimer’s disease is associated with cerebrospinal fluid neurofilament light in a representative sample of cognitively unimpaired 70-year olds. J Gerontol A Biol Sci Med Sci. 2021;76:983–90. 33512503 10.1093/gerona/glab030 PMC8140047

[8] Porter T, Burnham SC, Milicic L, Savage G, Maruff P, Lim YY, et al. Utility of an Alzheimer’s disease risk-weighted polygenic risk score for predicting rates of cognitive decline in preclinical Alzheimer’s disease: a prospective longitudinal study. J Alzheimers Dis. 2018;66:1193–211. 30412495 10.3233/JAD-180713

[9] Xu Y, Vasiljevic E, Deming YK, Jonaitis EM, Koscik RL, Van Hulle CA, et al. Effect of pathway-specific polygenic risk scores for Alzheimer’s disease (AD) on rate of change in cognitive function and AD-related biomarkers among asymptomatic individuals. J Alzheimers Dis. 2023;94:1587–605. 37482996 10.3233/JAD-230097 PMC10468904

[10] Lupton MK, Strike L, Hansell NK, Wen W, Mather KA, Armstrong NJ, et al. The effect of increased genetic risk for Alzheimer’s disease on hippocampal and amygdala volume. Neurobiol Aging. 2016;40:68–77. 26973105 10.1016/j.neurobiolaging.2015.12.023 PMC4883003

[11] Chaudhury S, Brookes KJ, Patel T, Fallows A, Guetta-Baranes T, Turton JC, et al. Alzheimer’s disease polygenic risk score as a predictor of conversion from mild-cognitive impairment. Transl Psychiatry. 2019;9:154. 31127079 10.1038/s41398-019-0485-7 PMC6534556

[12] Adams HHH, de Bruijn RFAG, Hofman A, Uitterlinden AG, van Duijn CM, Vernooij MW, et al. Genetic risk of neurodegenerative diseases is associated with mild cognitive impairment and conversion to dementia. Alzheimer’s & Dementia. 2015;11:1277–85. 10.1016/j.jalz.2014.12.008 25916564

[13] Harrison JR, Foley SF, Baker E, Bracher-Smith M, Holmans P, Stergiakouli E, et al. Pathway-specific polygenic scores for Alzheimer’s disease are associated with changes in brain structure in younger and older adults. Brain Communications. 2023;5:fcad229. 37744023 10.1093/braincomms/fcad229 PMC10517196

[14] Alzheimer’, s Association. 2014 Alzheimer’s disease facts and figures. Alzheimers Dement. 2014;10:e47–92. 10.1016/j.jalz.2014.02.001 24818261

[15] Snyder HM, Asthana S, Bain L, Brinton R, Craft S, Dubal DB, et al. Sex biology contributions to vulnerability to Alzheimer’s disease: a think tank convened by the Women’s Alzheimer’s Research Initiative. Alzheimer’s & Dementia. 2016;12:1186–96. 10.1016/j.jalz.2016.08.004 PMC10341380 27692800

[16] Dubal DB. Sex difference in Alzheimer’s disease: An updated, balanced and emerging perspective on differing vulnerabilities. Lanzenberger R, Kranz GS, Savic I, editors. Handb Clin Neurol. 2020;175:261–73. 10.1016/B978-0-444-64123-6.00018-7 33008530

[17] Buckley RF, Mormino EC, Amariglio RE, Properzi MJ, Rabin JS, Lim YY, et al. Sex, amyloid, and *APOE* ε4 and risk of cognitive decline in preclinical Alzheimer’s disease: findings from three well-characterized cohorts. Alzheimer’s & Dementia. 2018;14:1193–203. 10.1016/j.jalz.2018.04.010 PMC6131023 29803541

[18] Altmann A, Tian L, Henderson VW, Greicius MD. Sex modifies the APOE-related risk of developing Alzheimer disease: sex andAPOE-related AD risk. Ann Neurol. 2014;75:563–73. 24623176 10.1002/ana.24135 PMC4117990

[19] Molinuevo JL, Gramunt N, Gispert JD, Fauria K, Esteller M, Minguillon C, et al. The ALFA project: a research platform to identify early pathophysiological features of Alzheimer’s disease. Alzheimer’s & Dementia: Translational Research & Clinical Interventions. 2016;2:82–92. 29067295 10.1016/j.trci.2016.02.003 PMC5644283

[20] Vilor-Tejedor N, Genius P, Rodríguez-Fernández B, Minguillón C, Sadeghi I, González-Escalante A, et al. Genetic characterization of the ALFA study: uncovering genetic profiles in the Alzheimer’s continuum. Alzheimers Dement. 2023. 10.1002/alz.13537. 38088508 10.1002/alz.13537 PMC10984507

[21] Milà-Alomà M, Salvadó G, Gispert JD, Vilor-Tejedor N, Grau-Rivera O, Sala-Vila A et al. Amyloid-β, tau, synaptic, neurodegeneration and glial biomarkers in the preclinical stage of the Alzheimer’s continuum. Alzheimer’s % Dementia [Internet]. 2020; Available from: https://pubmed.ncbi.nlm.nih.gov/32573951/10.1002/alz.12131 PMC7586814 32573951

[22] Papp KV, Rentz DM, Orlovsky I, Sperling RA, Mormino EC. Optimizing the preclinical Alzheimer’s cognitive composite with semantic processing: the PACC5. Alzheimers Dement (N Y). 2017;3:668–77. 29264389 10.1016/j.trci.2017.10.004 PMC5726754

[23] Blauwendraat C, Faghri F, Pihlstrom L, Geiger JT, Elbaz A, Lesage S, et al. NeuroChip, an updated version of the NeuroX genotyping platform to rapidly screen for variants associated with neurological diseases. Neurobiol Aging. 2017;57:247.e9-247.e13. 28602509 10.1016/j.neurobiolaging.2017.05.009 PMC5534378

[24] Das S, Forer L, Schönherr S, Sidore C, Locke AE, Kwong A, et al. Next-generation genotype imputation service and methods. Nat Genet. 2016;48:1284–7. 27571263 10.1038/ng.3656 PMC5157836

[25] Choi SW, O’Reilly PF. PRSice-2: Polygenic Risk Score software for biobank-scale data. Gigascience [Internet]. 2019;8. Available from: 10.1093/gigascience/giz082 10.1093/gigascience/giz082 PMC6629542 31307061

[26] Tesi N, van der Lee S, Hulsman M, Holstege H, Reinders MJT. snpXplorer: a web application to explore human SNP-associations and annotate SNP-sets. Nucleic Acids Res. 2021;49:W603-12. 34048563 10.1093/nar/gkab410 PMC8262737

[27] Kivipelto M, Ngandu T, Laatikainen T, Winblad B, Soininen H, Tuomilehto J. Risk score for the prediction of dementia risk in 20 years among middle aged people: a longitudinal, population-based study. Lancet Neurol. 2006;5:735–41. 16914401 10.1016/S1474-4422(06)70537-3

[28] Salvadó G, Brugulat-Serrat A, Sudre CH, Grau-Rivera O, Suárez-Calvet M, Falcon C, et al. Spatial patterns of white matter hyperintensities associated with Alzheimer’s disease risk factors in a cognitively healthy middle-aged cohort. Alzheimers Res Ther. 2019;11:12. 30678723 10.1186/s13195-018-0460-1 PMC6346579

[29] Iglesias JE, Augustinack JC, Nguyen K, Player CM, Player A, Wright M, et al. A computational atlas of the hippocampal formation using ex vivo, ultra-high resolution MRI: application to adaptive segmentation of in vivo MRI. Neuroimage. 2015;115:117–37. 25936807 10.1016/j.neuroimage.2015.04.042 PMC4461537

[30] Dickerson BC, Feczko E, Augustinack JC, Pacheco J, Morris JC, Fischl B, et al. <article-title update="added">Differential effects of aging and Alzheimer’s disease on medial temporal lobe cortical thickness and surface area. Neurobiol Aging. 2009;30:432–40. 17869384 10.1016/j.neurobiolaging.2007.07.022 PMC3703585

[31] Levine DA, Gross AL, Briceño EM, Tilton N, Giordani BJ, Sussman JB, et al. Sex differences in cognitive decline among US adults. JAMA Netw Open. 2021;4:e210169. 33630089 10.1001/jamanetworkopen.2021.0169 PMC7907956

[32] Martins IJ, Berger T, Sharman MJ, Verdile G, Fuller SJ, Martins RN. Cholesterol metabolism and transport in the pathogenesis of Alzheimer’s disease. J Neurochem. 2009;111:1275–308. 20050287 10.1111/j.1471-4159.2009.06408.x

[33] Cuenca-Bermejo L, Prinetti A, Kublickiene K, Raparelli V, Kautzky-Willer A, Norris CM, et al. Fundamental neurochemistry review: old brain stories - influence of age and sex on the neurodegeneration-associated lipid changes. J Neurochem. 2023;166:427–52. 37161795 10.1111/jnc.15834

[34] Laws KR, Irvine K, Gale TM. Sex differences in cognitive impairment in Alzheimer’s disease. World J Psychiatry. 2016;6:54–65. 27014598 10.5498/wjp.v6.i1.54 PMC4804268

[35] Georgakis MK, Kalogirou EI, Diamantaras A-A, Daskalopoulou SS, Munro CA, Lyketsos CG, et al. Age at menopause and duration of reproductive period in association with dementia and cognitive function: a systematic review and meta-analysis. Psychoneuroendocrinology. 2016;73:224–43. 27543884 10.1016/j.psyneuen.2016.08.003

[36] Plaza-Rosales I, Brunetti E, Montefusco-Siegmund R, Madariaga S, Hafelin R, Ponce DP, et al. Visual-spatial processing impairment in the occipital-frontal connectivity network at early stages of Alzheimer’s disease. Front Aging Neurosci. 2023;15:1097577. 36845655 10.3389/fnagi.2023.1097577 PMC9947357

[37] Walters S, Contreras AG, Eissman JM, Mukherjee S, Lee ML, Choi S-E, et al. Associations of sex, race, and Apolipoprotein E alleles with multiple domains of cognition among older adults. JAMA Neurol. 2023;80:929–39. 37459083 10.1001/jamaneurol.2023.2169 PMC10352930

[38] Ferretti MT, Ding H, Au R, Liu C, Devine S, Auerbach S, et al. Maximizing utility of neuropsychological measures in sex-specific predictive models of incident Alzheimer’s disease in the Framingham Heart Study. Alzheimer’s & Dementia. 2024;20:1112–22. 10.1002/alz.13500 PMC10917035 37882354

[39] Ossenkoppele R, Pijnenburg YAL, Perry DC, Cohn-Sheehy BI, Scheltens NME, Vogel JW, et al. The behavioural/dysexecutive variant of Alzheimer’s disease: clinical, neuroimaging and pathological features. Brain. 2015;138:2732–49. 26141491 10.1093/brain/awv191 PMC4623840

[40] Peng G, Wang J, Feng Z, Liu P, Zhang Y, He F, et al. Clinical and neuroimaging differences between posterior cortical atrophy and typical amnestic Alzheimer’s disease patients at an early disease stage. Sci Rep. 2016;6:29372. 27377199 10.1038/srep29372 PMC4932506

[41] Eissman JM, Dumitrescu L, Mahoney ER, Smith AN, Mukherjee S, Lee ML, et al. Sex differences in the genetic architecture of cognitive resilience to Alzheimer’s disease. Brain. 2022;145:2541–54. 35552371 10.1093/brain/awac177 PMC9337804

[42] Ma H, Shi Z, Kim M, Liu B, Smith PJ, Liu Y, et al. Disentangling sex-dependent effects of APOE on diverse trajectories of cognitive decline in Alzheimer’s disease. Neuroimage. 2024;292:120609. 38614371 10.1016/j.neuroimage.2024.120609 PMC11069285

[43] Sánchez-Benavides G, Grau-Rivera O, Suárez-Calvet M, Minguillon C, Cacciaglia R, Gramunt N, et al. Brain and cognitive correlates of subjective cognitive decline-plus features in a population-based cohort. Alzheimers Res Ther. 2018;10:123. 30572953 10.1186/s13195-018-0449-9 PMC6302483

