# Supplementary figures and images for: Sex-specific early cognitive changes are linked to global and pathway-specific genetic risk for Alzheimer’s disease in at-risk individuals
Source: Biol Sex Differ. 2026 Feb 17;17:56. doi: 10.1186/s13293-025-00800-w (PMC13014877; doi:10.1186/s13293-025-00800-w)

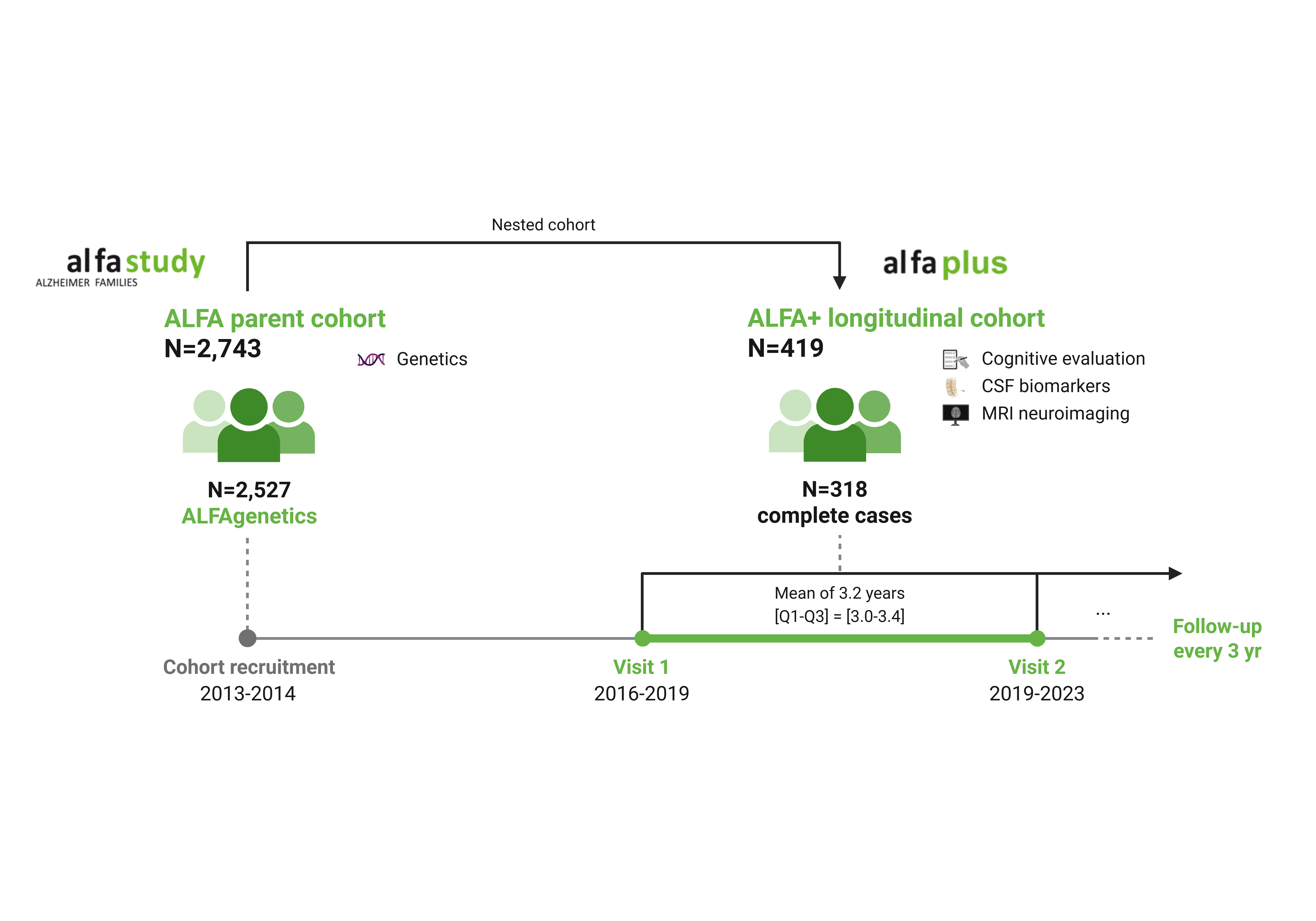

Supplement: Supplementary file 1 — Supplementary Material 1 [file 13293_2025_800_MOESM1_ESM.zip › Supplementary Material Revision/Supplementary Figure 1.png]

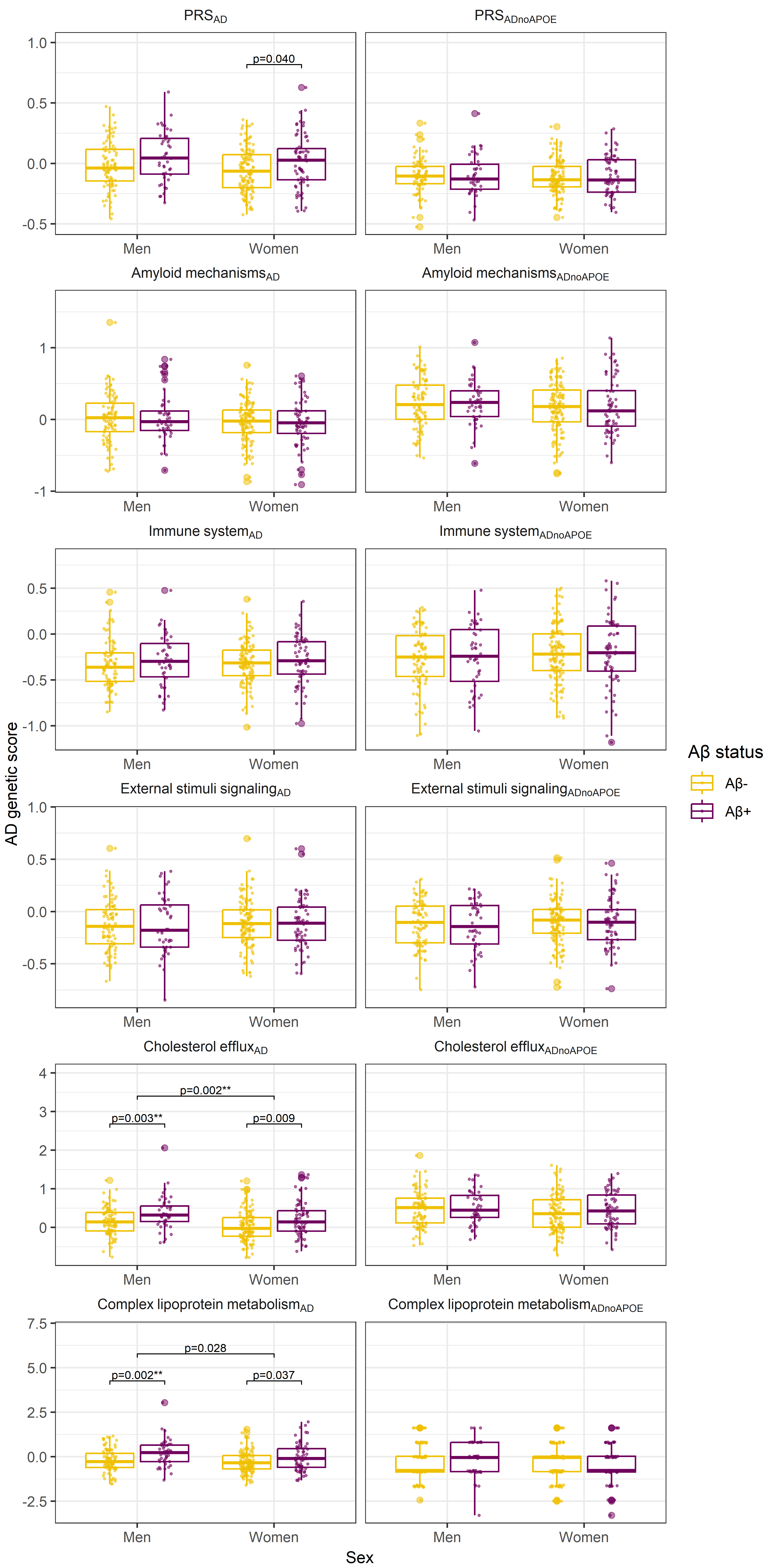

Supplement: Supplementary file 1 — Supplementary Material 1 [file 13293_2025_800_MOESM1_ESM.zip › Supplementary Material Revision/Supplementary Figure 2.png]
